# Label-free metabolic imaging and energy costs in Chlamydomonas

**DOI:** 10.1140/epje/s10189-025-00499-y

**Published:** 2025-07-11

**Authors:** Martine Boccara, Katia Wostrikoff, Benjamin Bailleuil, Claude Boccara

**Affiliations:** 1 Institut Langevin, ESPCI Paris, PSL Research University, CNRS UMR 7587, 1 Rue Jussieu, 75005 Paris, France; 2 ISYEB, Muséum National d’Histoire Naturelle, CNRS, Sorbonne Université, EPHE, Université Des Antilles, 57 Rue Cuvier, 75005 Paris, France; 3 Institut de Biologie Physicochimique, UMR 7141 CNRS / Sorbonne Université, 13 Rue Pierre et Marie Curie, 75005 Paris, France

## Abstract

We developed a label-free optical microscopy method to study movements of different frequencies and amplitudes within a cell. We use optical transmission tomography (OTT) that operates in transmission, and we record the changes of signal values of all the pixels of movies taken for a few seconds (dynamic signal). This signal is a metabolic signal in algae as it decreased in the presence of photosystem II inhibitors or when samples were illuminated at wavelengths where the photoreceptors are poorly operative. We used as model organism Chlamydomonas for which mutants are available. We used a mutant deleted of the chloroplastic gene encoding the large subunit of the Rubisco, *ΔrbcL*. This mutant is unable to fix atmospheric CO_2_ and is devoid of pyrenoid. We compared the dynamic signal between wild-type strain and *ΔrbcL* mutant of Chlamydomonas grown in dark condition and found it to be 5 to 10 times higher. This mutant overproduced starch, and we tempted to associate the metabolic signal to the cost in ATP_eq_ consumption for building starch. The method is easy to implement and could be very valuable for studies of phytoplankton in situ or virus-infected cells.

**Graphical abstract:**

**Figure:**
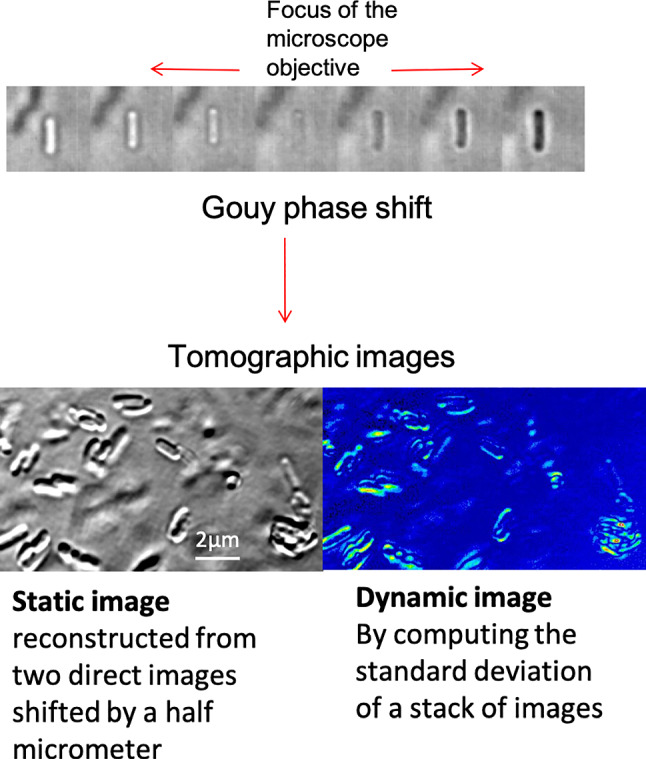
This graphical abstract illustrates the Gouy phase shift showing destructive and constructive interferences. With thisphase shift we generate two types of tomographic images: the static image informs for intracellular structures and thedynamic image provides information on the metabolic activity of the cell (all images are the bacteria Escherichia coli)

**Supplementary Information:**

The online version contains supplementary material available at 10.1140/epje/s10189-025-00499-y.

## Introduction

The microscopic observation in real time of cell metabolism is important in evolutionary studies to compare the energetic cost of different evolution paths [23]. Metabolic changes are a common feature of cancer cells, which needs to be evaluated for example in response to drugs. In the scope of global warming, it would also be more and more necessary to estimate physiological cellular responses from aquatic microbial communities in different latitudes.

ATP‐generating pathways in mammalian metabolism can be accurately measured in heterotrophic cells such as mammalian cell culture using commercial kits measuring by colorimetry the ratio NAD^+^/NADH or the rates of oxygen consumption and extracellular acidification [7]. Two-photon fluorescence microscopy methods have shown their efficiency to decipher physiological changes, using either the green fluorescent protein or taking advantage of autofluorescent molecules such as NAD(P)H present in all cells [2, 26, 27]**.**

We recently described a new label-free interferometric microscopy method which is non-invasive and non-destructive and which quantifies the metabolic activity of a cell [3, 19]. The setup is simple and can be achieved with commercially available cheap microscopes [4]. The method is label-free as the contrast is due to interferences between the illuminating beam (light-emitting diode, LED) and the one scattered by cell sub-micrometre structures (supplementary information (SI) fig.S1). Indeed, the scattered beam, collected by the microscope objective, is affected by the Gouy phase shift that takes place close to its focus [10, 11]. The method delivers a tomographic image either using a static mode to show the morphology of the biological sample or with a dynamic mode, which highlights the metabolic contrast within a cell.

We applied successfully this method to diatoms under environmental stresses (iron or phosphate deficiency) [3]. The cells were immobilized in agar, and a film was taken for a few seconds. We then computed the standard deviation of each pixel of the stack. We were able to show in diatoms that the detected dynamic signal was indeed a metabolic signal as it was dependent on photosynthetic activity (signal values dependent of the LED wavelength and of the use of PSII inhibitors) [3].

Apelian et al. [1] previously showed in mammalian cells that the recorded dynamic signal in liver cells was linked to the glycolytic pathway; indeed, it was inhibited by 2-deoxyglucose in these heterotrophic cells. We were interested to make a step further by correlating the dynamic signal within a cell with the energy consumption (expressed in ATP equivalent (ATP_eq_)) for building macromolecules. For this purpose, we used an autotrophic model organism Chlamydomonas for which mutants are easy to obtain [13]. Indeed, mutants in photosynthesis or light perception have been obtained for this unicellular green alga. In addition, Chlamydomonas has the ability to grow non-photosynthetically with acetate as sole carbon source.

Chlamydomonas captures CO_2_ thanks to the pyrenoid, a liquid-like organelle within the chloroplast, which contains the enzyme ribulose biphosphate carboxylase (Rubisco) a key enzyme of the Calvin cycle [21]. CO_2_ fixation leads to glucose, which is stored as starch granules. In our study, we used a mutant deleted of the chloroplastic gene encoding the large subunit of the Rubisco, *ΔrbcL*. This mutant is unable to fix atmospheric CO_2_ and is devoid of pyrenoid [6, 16].

Here we computed the dynamic signal in the wild-type strain globally and described its distribution microscopically. We compared the wild-type strain dynamic signal with that of *ΔrbcL* and find a higher signal in the mutant with a different microscopic distribution. Finally, we estimated the energy consumption in ATP_eq_ to build a starch molecule of one thousand units, in *ΔrbcL* mutant. The comparison between wild-type and *ΔrbcL* mutant dynamic signal when grown in dark condition showed an agreement with the observation of an accumulation of starch in the same mutant described by Saint-Sorny et al*.* [22].

## Material and methods

### Media, culture conditions, and strains

*Chlamydomonas reinhardtii*, wild-type strain and mutants, were grown in 500-mL flasks and agitated on a gyratory shaker (120 rpm) at 25 °C under continuous light of 10 or 30 μmol photons m^−2^ s^−1^ (for mixotrophic and photosynthetic growth, respectively). Medium containing tris–acetate phosphate (TAP) or lacking acetate (MIN) was used for heterotrophic or phototrophic growth, respectively. The wild-type strain used in these experiments is T222 + , a 137c-derived reference strain (CC-5101; https://www.chlamycollection.org/) [9]. The construction of the RuBisCo less mutant (*ΔrbcL*) has been described in [16]. The eyeless mutant is the eye1-1 mutant (CC-1101 strain) [14]**.** Cells were collected by centrifugation and mixed with melted 1% agar (42 °C) in culture medium and immediately observed after solidification (20 °C).

### Dynamic cell imaging using full-field optical transmission tomography (FFOTT)

To follow the movement of internal structures within *Chlamydomonas* at the pixel level, we used a new experimental approach, called dynamic full-field optical transmission tomography (D-FFOTT) that is described in detail in Mazlin et al. (2022) [19] and presented in supplementary information (SI, fig.S1). In brief, the sample is illuminated in transmission by the incoherent light emitted by a LED; this beam is partially scattered by the sample structure and partially transmitted. Both beams propagate along the same path and interfere with a phase shift (known as the Gouy phase shift) that depends on the relative position of the scatterer and of the focus of the objective. The time series of the signal detected on each pixel of the camera (Photonfocus A1024B) is collected (SI, fig. S1c). To follow the evolution of signal over time, we took a stack of typically 100 images lasting a few seconds (frequency 20 images s^–1^).

We used a few seconds illumination to avoid light saturation of photosynthesis. Furthermore, with this short time illumination we did not noticed any effect on dynamic signal up to 1000 μmol photons m^–2^ s^–1^.

A fragment of agar including the cells (about 10 cells per field of view) was observed at room temperature for a few minutes as we chose different fields and took stacks of 100 frames. Experiments were repeated at least 3 times for any conditions (10 stacks per strain per experiment). For cells grown in the dark, the room was kept in dark with a red light.

The transmission image is then analysed with the open-source platform Fiji [24]. Histogram profiles of the standard deviation of signal intensity of each pixel were obtained, and the number of pixels above noise level was computed as described in [3]. All the values of different stacks from the same condition were visualized by boxplot, and comparison between samples was made using a Wilcoxon test.

In addition to this dynamic mode and to correlate the standard deviation image with cell structures (static FFOTT mode), we subtracted direct images at different depths (distant by 0.5 μm) which suppresses the background and enhances the light generated within from the depth of field, thus producing an optical section.

### Birefringence

To observe birefringence in Chlamydomonas, we illuminated the sample with a circular polarization and made two measurements along two linear polarizations at 45° from each other. The polarization set up was coupled to the interferometric set up.

## Results

### The dynamic signal in wild-type Chlamydomonas depends on photosynthetic activity

We first studied Chlamydomonas wild-type strain either in stationary or exponential phases (10 or 4 10^6^ cells mL^−1^) using OTT method (SI, fig. S1). The cells grown in TAP liquid medium with a light intensity of 10 μmoles of photons m^−2^ s^−1^ were collected by centrifugation. To reveal scatterers motions within the cells, the algae were immobilized in TAP medium with 1% agar. After solidification, a fragment of agar was deposed under the objective and a movie of about 5 s recorded. The computation of the number of pixels whose values are changing during the movie is obtained by measuring the standard deviation of each pixel of the stack of images. We computed then the dynamic signal, which is the sum of signals above background as already described [3]. The signal corresponded to an average of ten cells per field.

**Fig. 1:**
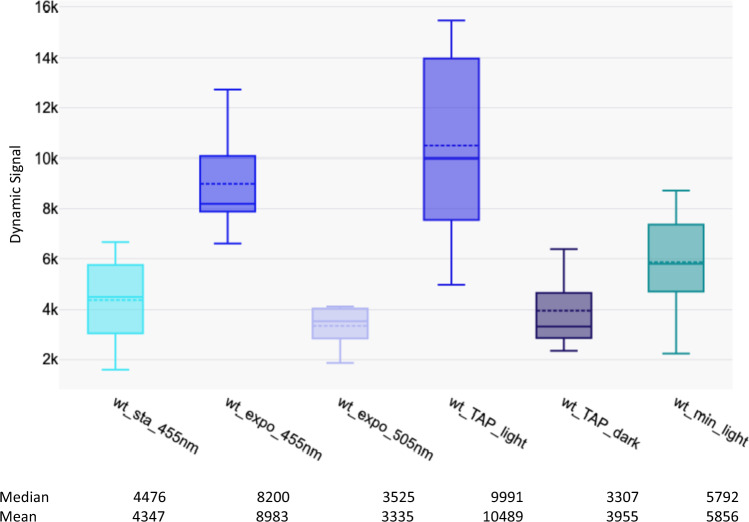
Dynamic signal in wild-type cells. (Left) Box plots of dynamic signal (number of pixels whose values are changing during the movie) in wild type in different conditions (sta for stationary growth and expo for exponential growth in TAP medium with 10 μmoles of photons m^−2^ s^−1^). (Right) Box plots of wild-type strain exponentially grown in TAP medium and light, in TAP medium in dark, and in min medium with bubbling CO_2_ and light (30μmoles of photons m^−2^ s^−1^; wt_TAP_light is significantly different from wt_TAP_dark at p < 0.05 (p = 0.00012) and is significantly different from wt_min_light at p < 0.05 (p = 0.00038). The measurements corresponded to different fields of the sample. Median values are indicated by continuous lines and mean by dotted lines. Experiments were repeated at least three times, and results of one experiment are presented

The signal intensity was two times higher in exponential growth than in stationary phase (Fig. 1). We thus did all the following experiments with exponentially growing cells. We then illuminated Chlamydomonas with a green LED (505 nm) a spectral region where pigments poorly absorb and photosystems are little excited [20]. We observed that the dynamic signal was reduced three times at 505 nm than when cells were illuminated with the 455-nm LED suggesting that the dynamic signal in the wild-type cells depended mainly on photosynthetic activity (Fig. 1). To confirm this observation, we grew the cells in dark with acetate or in autotrophic conditions. As expected, the signal was reduced three times in dark condition. In autotrophic conditions, the signal was lower than in mixotrophic condition possibly because there was no acclimation step for this condition [12].

### The strongest dynamic signal in the wild type is located in the eyespot

**Fig. 2:**
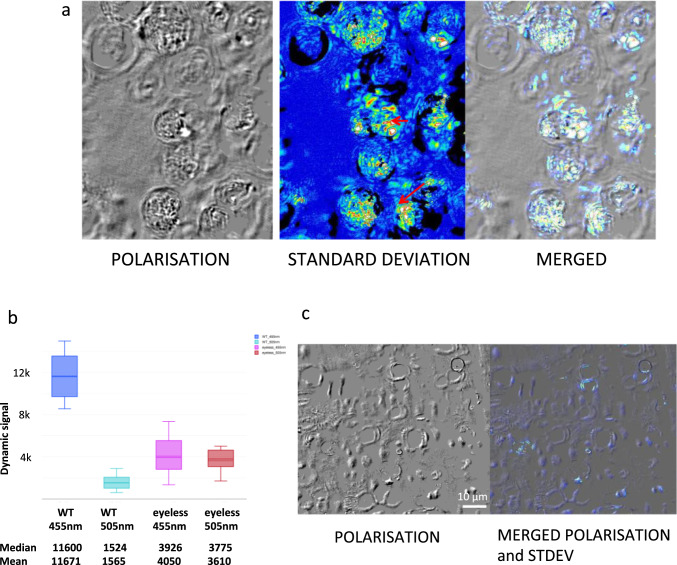
Dynamic signal in the wild-type strain is mostly located in the eyespot. **a** From the left: polarization images of wild-type cells**,** standard deviation image (artificial colours) of a movie taken without analyser (no polarization) of the same field; merged images of polarization and standard deviation. **b** Box plot of dynamic signals of wild-type strain compared to an eyeless mutant at 455 and 505 nm; median and mean are indicated. **c** Polarization image of eyeless mutant (left) and merged images of polarization and standard deviation (right)

We observed in the wild-type strain a movement in the periphery of the cells as the film was running (SI, movie S1). This movement translated in a highly dynamic spot on standard deviation image (Fig. 2a). The flagella that would be supposed to be the most dynamic structures of the cells were immobilized by agar [23]. We thus hypothesized that this spot could correspond to the eyespot of *Chlamydomonas*. The eyespot is the organ that detects the direction of the incoming light and subsequently leads to the movement of flagella [15]. The eyespot is a dynamic birefringent structure [17, 28]. Combining polarization and interferometry, we were able to evidence a strong dynamic signal (standard deviation) merged with the birefringent signal (arrows) (Fig. 2a). To confirm this observation, we analysed the dynamic signal in an eyeless mutant [18]. This mutant did not produce any bright spots in polarization and only few birefringent signals in the cell wall. The dynamic signal at 455 nm was three times reduced compared to that of the wild-type strain (Fig. 2b). In addition, the dynamic signal was mostly distributed in the cell wall and was very different from the distribution in wild-type *Chlamydomonas* (Fig. 2c). We concluded that in the wild type most of the dynamic signal is due to strong or weak signals of the eyespot.

### *ΔrbcL* cells produce a higher dynamic signal than wild-type cells and differently distributed

**Fig. 3:**
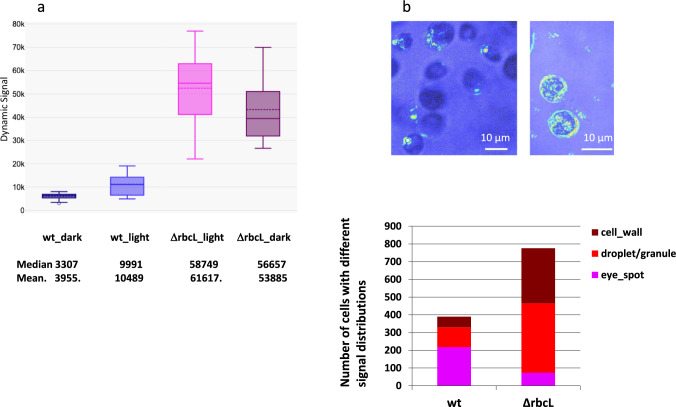
Dynamic signal is distributed differently in wild-type and *ΔrbcL* strains. **a** Dynamic signal in wild-type strain (left) and *ΔrbcL* mutant from seven independent stacks (50–100 cells). The wt is significantly different from ΔrbcL at p < 0.05 (p = 0.0003). The box plots of *ΔrbcL* grown in TAP medium either with light (10 μmoles of photons m^−2^ s.^−1^) or in dark were not significantly different (p = 0.25428). **b** Images of the dynamic signal distribution in wild-type strain (left) (SI, movie_S1) and of *ΔrbcL* mutant showing dynamic signal associated with irregular structures (droplets or granules) and with cell wall (right). **c** Distribution of the dynamic signal: in the wild-type strain, the signal is mostly located in the eye spot (≈50%), and in *ΔrbcL* mutant the dynamic signal is mostly associated with droplets or granules (≈60%) and less with the eye spot (≈10%)

We then compared the dynamic signal of *ΔrbcL* mutant and wild-type cells grown in mixotrophic condition. We observed a higher signal in *ΔrbcL* mutant about 3 to 6 times that of the wild-type strain suggesting a higher metabolic activity in the mutant (Fig. 3a). Furthermore, the dynamic signal was independent of photosynthetic activity, as it was not significantly reduced when illuminated with the 505-nm LED (SI, fig. S2).

To investigate the reasons for a higher dynamic signal in *ΔrbcL* mutant than in wild-type strain, we compared the dynamic signals of the wild-type strain (Fig. 1) and *ΔrbcL* mutant grown in dark condition (Fig. 3). We observed a 5 to 10 times higher signal in the mutant compared to wild type (p = 0.00044), and there was no significant difference (p = 0.25428) between *ΔrbcL* mutant grown with TAP medium in light and dark conditions (Fig. 3a).

We also observed a different distribution of the signal in the *ΔrbcL* mutant compared to wild type: in the wild-type strain, the signal was mostly located in the eye spot (≈50%) while in *ΔrbcL* mutant the dynamic signal was mostly located in droplet/granules structures (60%) and less in eye spot (≈10%) (figs. 3b, 3c and SI, Fig.S3).

### Correlation between the optical signal and metabolic activity in ΔrbcL mutant

*Chlamydomonas* as all living organisms requires energy for construction of molecules, and we were interested to associate the dynamic FFOTT signal to the cost in ATP_eq_ consumption for building starch. Saint-Sorny et al. [22] showed that the *ΔrbcL* mutant produces six times more starch than the wild type when grown in TAP medium with the same light conditions as the one we used. In agreement with this result, we observed by polarization that the *ΔrbcL* mutant exhibits an increase in typical “maltese crosses” corresponding to starch granules (SI. fig.S3, arrows) and two times increase in the number of dynamic irregular structures (droplet/granules, Fig. 3c).

The *ΔrbcL* mutant does not accumulate Rubisco and has thus a broken Calvin cycle. It cannot grow autotrophically, and acetate is used as carbon source (SI, fig, S4). Saint-Sorny et al. [22] studied the carbon metabolism of *ΔrbcL* mutant and showed net accumulation of the carboxylic acids malate and succinate suggesting an increase in the activity of the glyoxylate cycle as well as an increase in glucose-6 phosphate a starch synthesis precursor (SI, fig. S4).

We computed different metabolic steps involving ATP_eq_ (ATP equivalent or cell energy currency) consumption first, to make glucose-6P and then for building a starch molecule of 1000 glucose units (SI, fig. S4) in the *ΔrbcL* cell. We aimed at correlating the optical signal we quantified (Fig. 3a) with the activity of an anabolic pathway (starch biosynthesis). The *ΔrbcL* mutant growth relies only on the presence of acetate; indeed, we observed no significant differences between the dynamic signal of *ΔrbcL* mutant grown with light or dark conditions (Fig. 3a). So, the growth of *ΔrbcL* mutant is strictly heterotrophic. For acetate assimilation, 2 ATP and 1NADH are necessary per acetate. Acetate is a two-carbon molecule so 3 molecules of acetate are necessary to make one glucose molecule. The textbooks translate 1 NADH in 2.5 ATP_eq_; we thus estimated the number of ATP_eq_ necessary to make one molecule of glucose to be 13.5 ATP_eq_. For each glucose, one ATP is necessary to contribute to starch synthesis: to make a starch molecule of 1000 glucose units, 13.5 10^3^ATP + 10^3^ ATP = 14.5 10^3^ ATP_eq_ are necessary.

We observed that the wild-type strain grown in the dark produces a signal three times lower than in light condition. Indeed, most of the ATP needed are for flagella and eyespot movements; in support of that, we observed in the wild-type strain grown in mixotrophic condition a distribution of the dynamic signal mostly in the eye spot (about 50% of the signals) as the flagella are immobilized in agar. In the *ΔrbcL* mutant, only 10% of the signals are localized at the eyespot, confirming that photomechanical energy is redirected in the mutant.

We were not able to compute the ATP_eq_ of the wild-type strain in mixotrophic conditions as we could not determine how energy is distributed between heterotrophic and autotrophic growths. The wild type in dark condition does not accumulate starch like the *ΔrbcL* mutant suggesting that although mutated in a single gene it has a more complex phenotype than expected [22].

## Discussion

Here we revealed a dynamic signal within the photosynthetic microorganism *Chlamydomonas* and showed that this signal was related to cell metabolism. First, we asked how the signal is generated? We clearly showed in diatoms [3] and in *Chlamydomonas* wild type that the signal was related to the photosynthetic activity of the cells. Indeed, the signal decreased three times in the dark and when cells were illuminated at 505 nm a wavelength where very little pigments absorption is observed [20]. We compared the dynamic signals of wild-type strain and a pyrenoid mutant (*ΔrbcL*) unable to complete the Calvin cycle. We observed reproducibly a signal three to six times higher in the mutant grown with acetate as sole carbon source while the wild-type strain relies on acetate and light for its growth.

How could we relate an optical signal to the movement of scatterers within the cell? What kind of movement could we evidence? Light is sensed by photoreceptors located in the eyespot on the side of the cell [25]. The eyespot is a primitive visual system that regulates the activity of a motor apparatus, the flagella. We immobilized the cells in agar to avoid phototactic behaviour, where sensing the direction of light intensity is examined by the eyespot and transduced as an electromechanical signal to flagella leading cells to swim towards light. Wild-type cell observations showed often a localized dynamic signal that corresponds to the eyespot as highly dynamic, co-localized with birefringent structures and that disappears in an eyeless mutant.

If we can associate the dynamic signal to metabolic activity, it seems unlikely that it could correspond to ATP_eq_ generation as it is known that ATP is rapidly consumed and never stored as so. We may consider that the dynamic signal is more associated with ATP consumption either for scatterer movements such as the flagella or the eyespot in the wild type and/or for the storage of energy as macromolecules. Indeed, we observed also dynamic signal associated with chloroplastic structures (droplets/granules). Why these structures were found dynamic? We propose two hypotheses: the movement of these droplets in the cells could be possibly due to their association with cytoskeleton or probably due to the metabolite fluxes, for example, the formation of starch granules. We have explained previously the method that allowed to this conclusion when analysing the dynamic signal in lipid bodies in *Phaeodactylum* [3].

In the future, it would be interesting to combine microscopic analysis of films of phytoplankton collected in the oceans with the dynamic signal. The dynamic signal, which is easy to determine as shown here, could be an indicator of the metabolic status of these microorganisms in situ before undertaking metabolite analysis. The same method could also be applied to detect cancer cells, which are known to often rely on the glycolytic pathway for energy generation while normal cells are able to perform the oxidative phosphorylation pathway [5]. Finally, improvement in our set-up should be possible to study cells growing on glass support or in three dimensions and subjected to drugs or to viral infection.

## Supplementary Information

Below is the link to the electronic supplementary material

Supplementary file1 (PPTX 7752 KB)
