# Supplementary material for: Label-free metabolic imaging and energy costs in Chlamydomonas
Source: Eur Phys J E Soft Matter. 2025 Jul 11;48(6-7):38. doi: 10.1140/epje/s10189-025-00499-y (PMC12254062; doi:10.1140/epje/s10189-025-00499-y)

## Slide 1
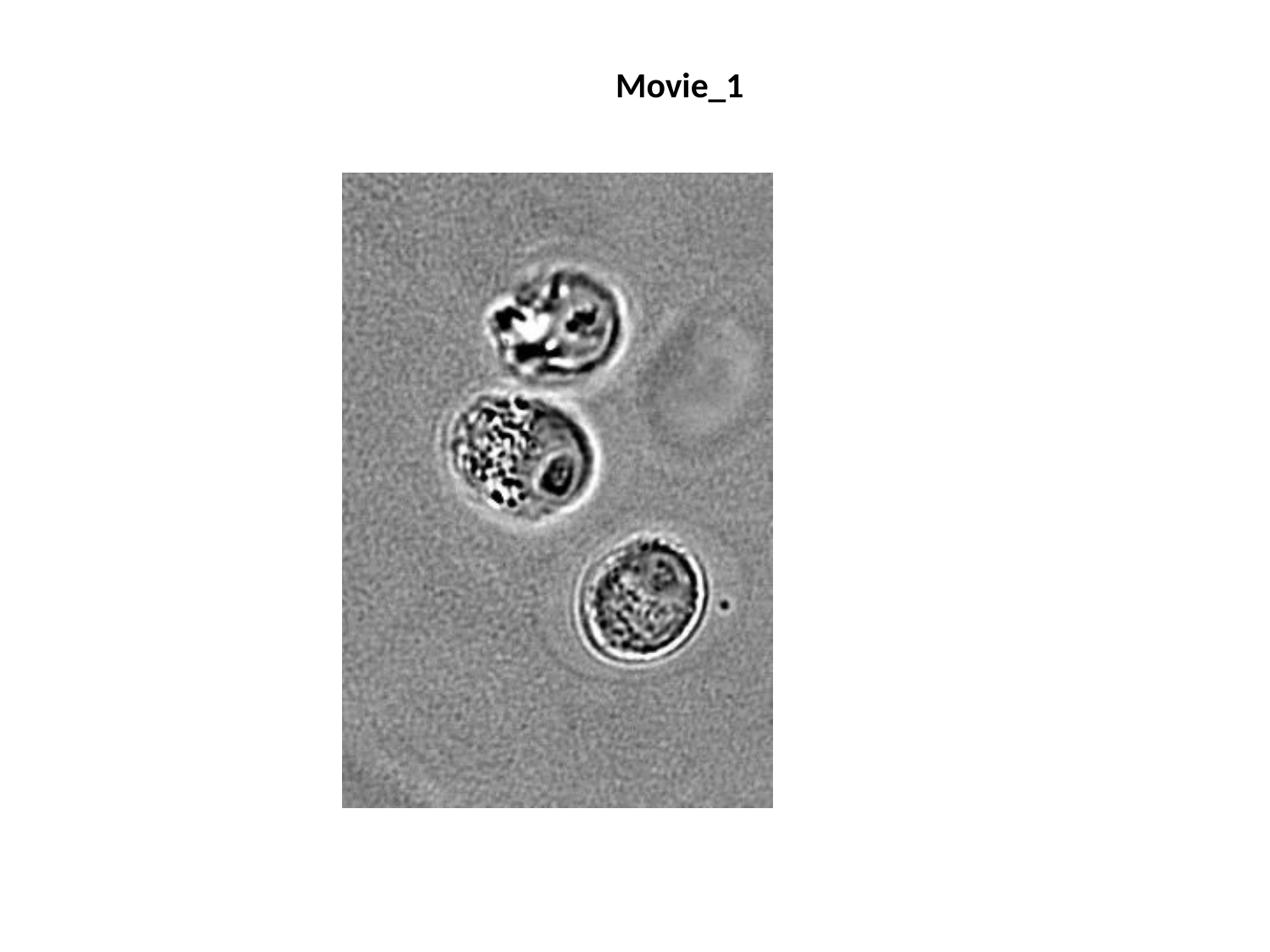

Movie_1

## Slide 2
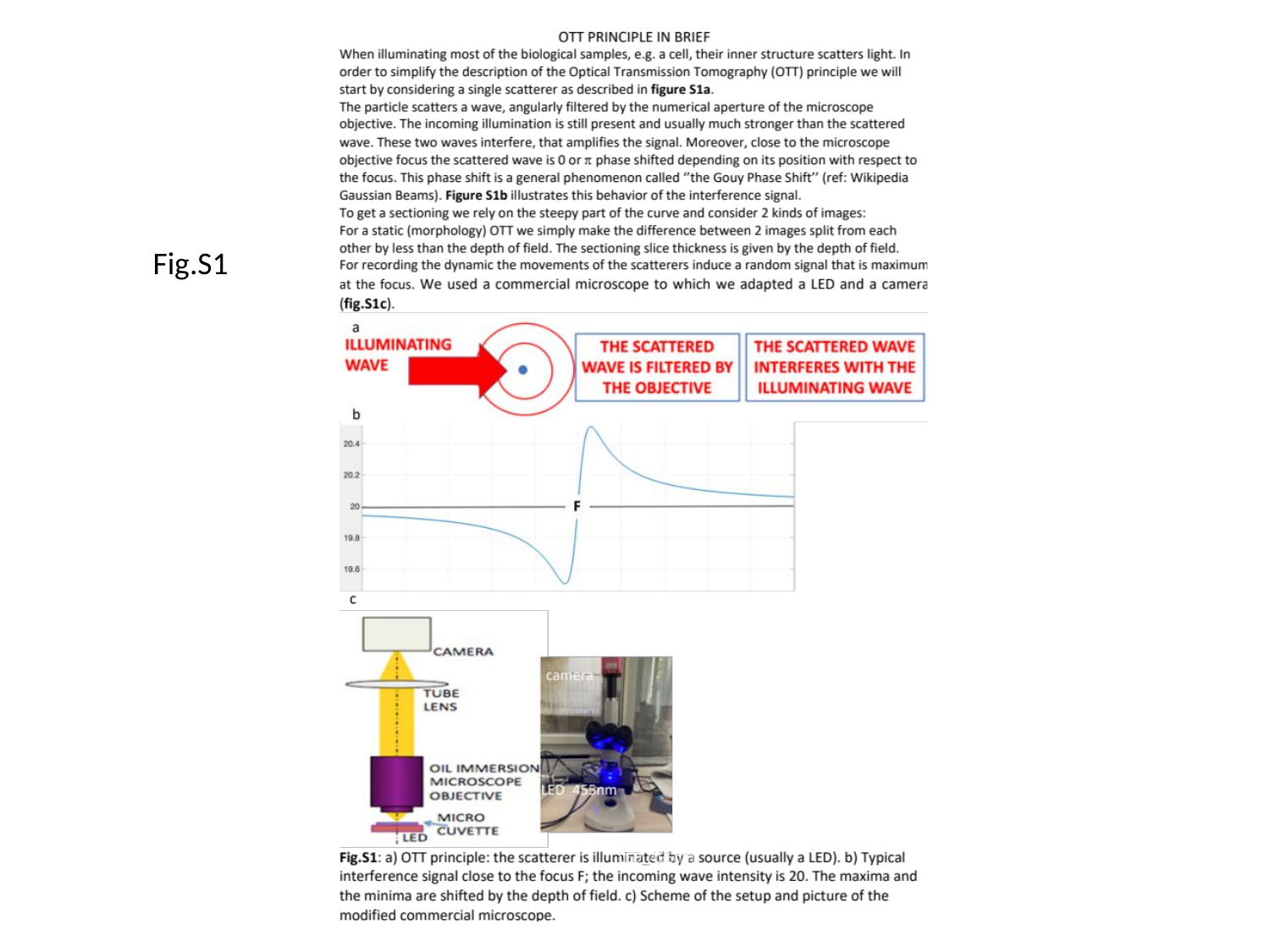

Fig.S1

## Slide 3
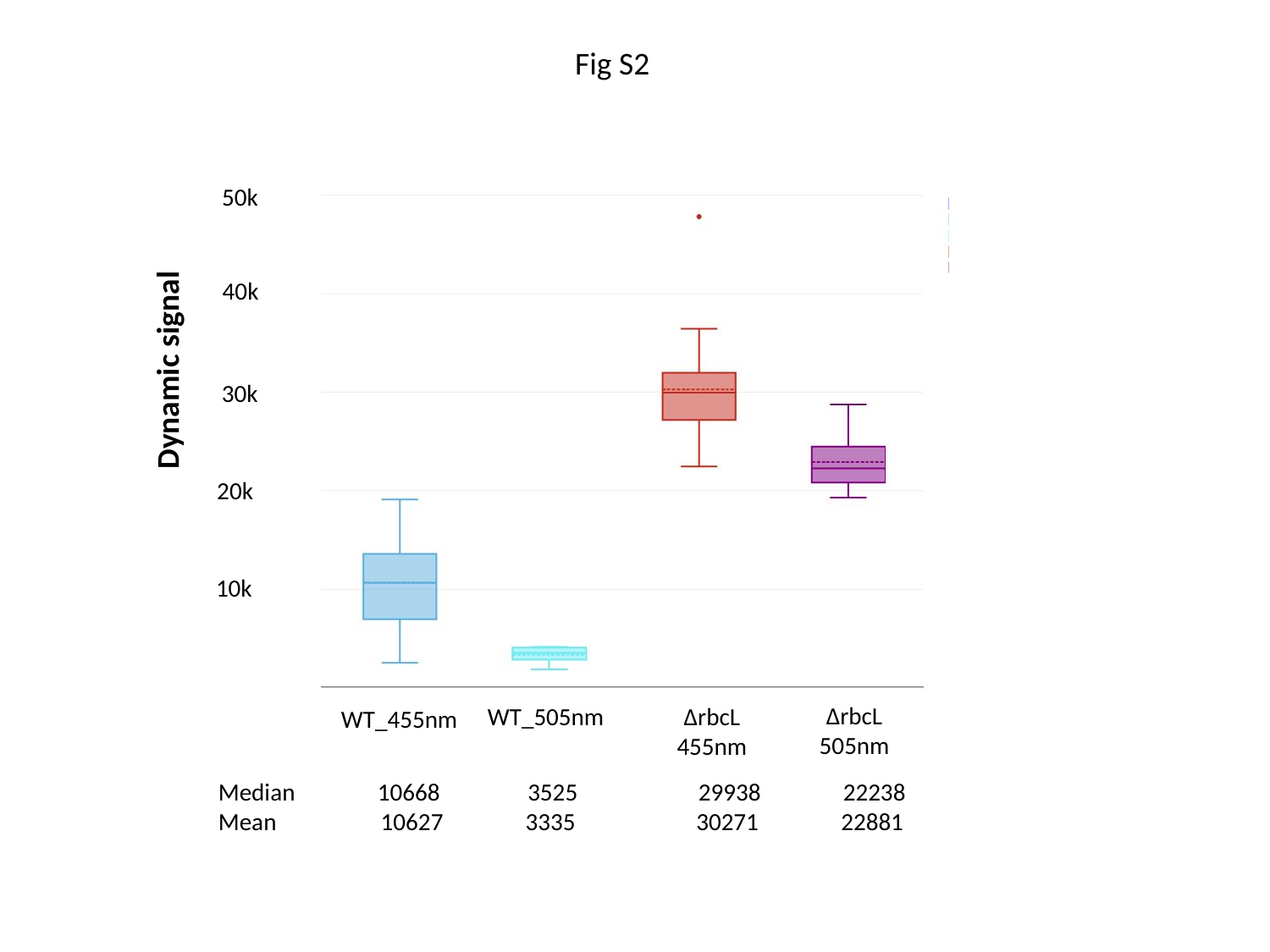

Fig S2
 50k
 40k
 30k
20k
10k
Dynamic signal
ΔrbcL
505nm
WT_505nm
ΔrbcL
455nm
WT_455nm
 Median 10668 3525 29938 22238
 Mean 10627 3335 30271 22881

## Slide 4
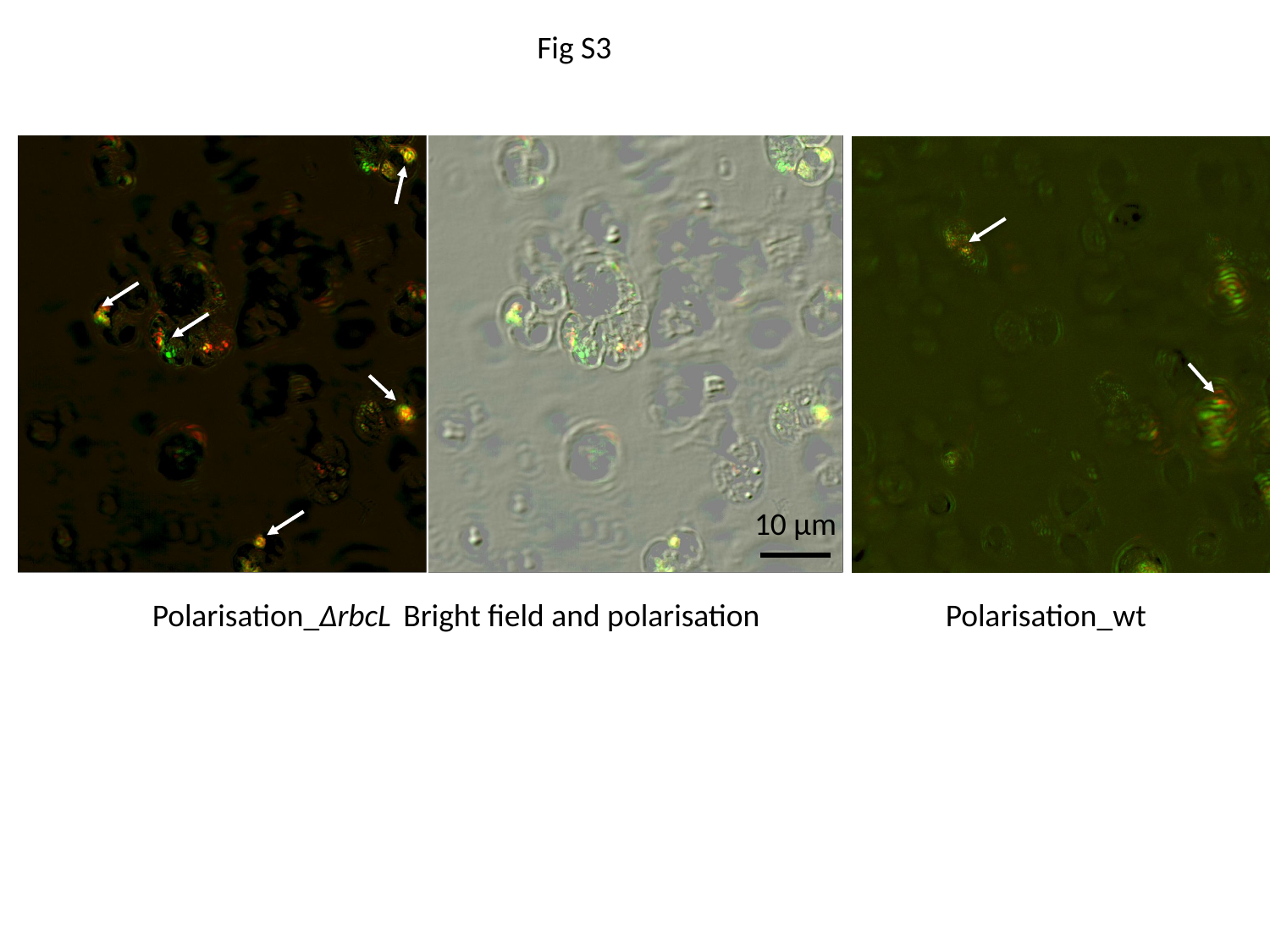

Fig S3
10 µm
Polarisation_ΔrbcL
Bright field and polarisation
Polarisation_wt

## Slide 5
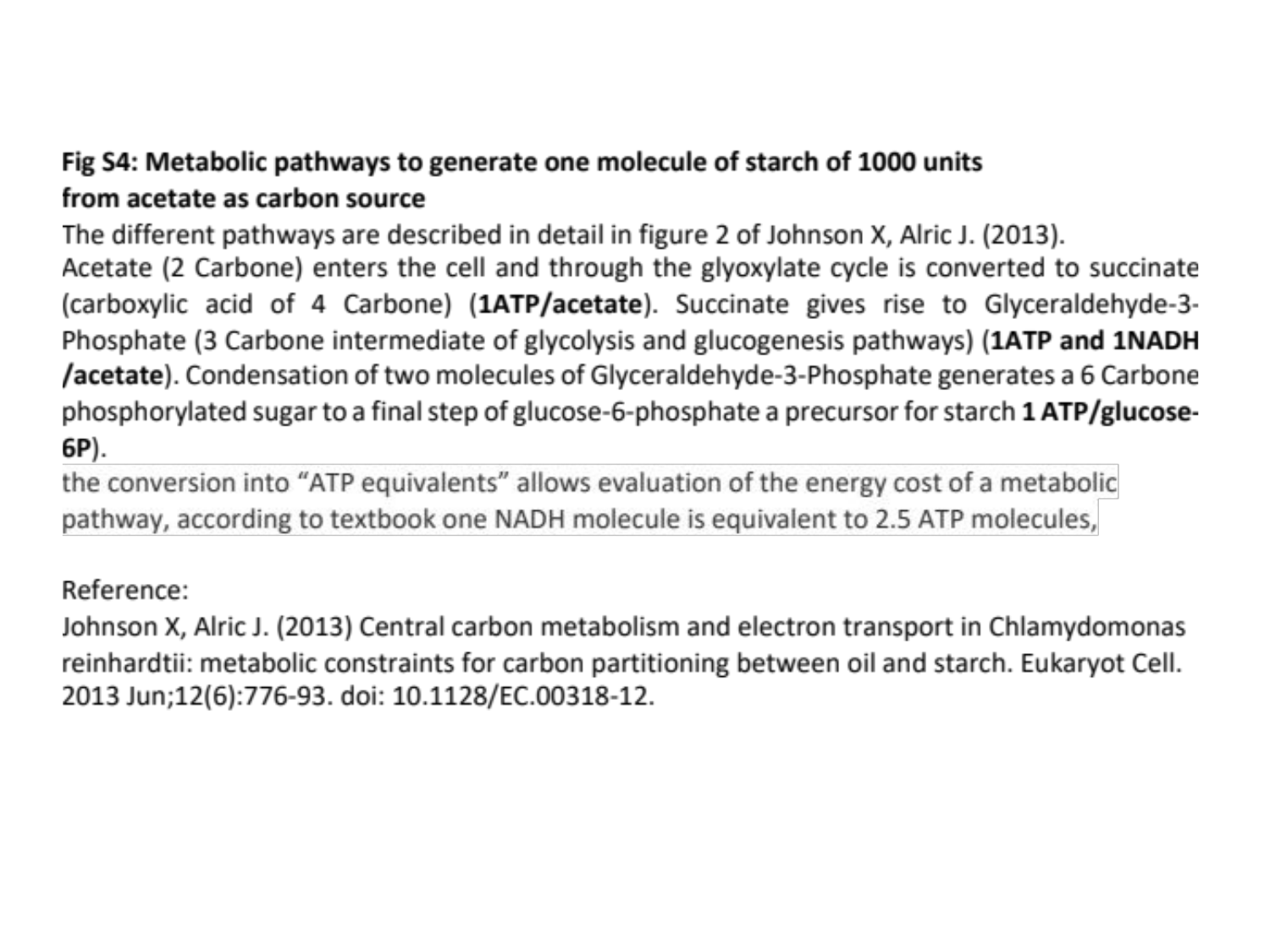

Supplement: Supplementary file 1 — Supplementary file1 (PPTX 7752 KB) [file 10189_2025_499_MOESM1_ESM.pptx]
